# Effects of *Orthonairovirus hazaraense* Nucleoprotein on Gene Expression Profiles in Infected Cells

**DOI:** 10.3390/v18010025

**Published:** 2025-12-23

**Authors:** Keisuke Ohta, Machiko Nishio

**Affiliations:** Department of Microbiology, School of Medicine, Wakayama Medical University, Wakayama 641-8509, Japan; mnishio@wakayama-med.ac.jp

**Keywords:** *Orthonairovirus hazaraense*, nucleoprotein, transcriptome

## Abstract

Nucleoprotein (N) of *Orthonairovirus hazaraense* (HAZV) not only encapsidates viral genomic RNA but also has the potential to regulate functions of host factors. To screen for host factors affected by HAZV N protein, we investigated the effects of HAZV N protein on the gene expression profile by transcriptome analysis of a HAZV-infected SW13 cell line constitutively expressing HAZV N protein (SW13/N). The gene expression profile of HAZV-infected SW13/N was quite different from that of HAZV-infected SW13 cells. Notably, HAZV infection up-regulated many immune-response-related genes in SW13 cells, but not in SW13/N. This clearly indicates the suppression of host immune response by N protein. Among immune-response-related genes, the myeloid differentiation factor 88 (*MyD88*) gene was uniquely down-regulated in SW13/N, despite its up-regulation by HAZV infection. Furthermore, MyD88 was also down-regulated at the protein level in SW13/N. N protein was also found to potentially down-regulate cell adhesion, cell cycle, apoptosis and cytoskeleton-related genes. HAZV N protein is suggested to have a great impact on the gene expression profile in infected cells. This is the first report of comprehensive analysis of host gene expression that is manipulated by nairovirus protein.

## 1. Introduction

*Orthonairovirus haemorrhagiae* (formerly known as Crimean-Congo hemorrhagic fever virus; CCHFV) and *Orthonairovirus hazaraense* (HAZV) are tick-borne viruses belonging to the *Orthonairovirus* genus of the *Nairoviridae* family in the order *Hareavirales* (http://talk.ictvvvonline.org/taxonomy (accessed on 10 November 2025)). CCHFV causes a severe hemorrhagic disease (CCHF) with a high rate of lethality (approximately 30%). CCHFV is classified as biosafety level (BSL) 4, although there is a report calling for downgrading of CCHFV research work to BSL 3 [1]. There are no approved medical countermeasures for CCHF, despite the high potential for its epidemic spread. HAZV, by contrast, is non-pathogenic to humans and can be handled in BSL 2 facilities. HAZV and CCHFV exhibit the same pathology in adult type I IFN receptor-knockout mice [2,3,4], although these viruses are different BSL pathogens. Furthermore, both HAZV and CCHFV have high pathogenicity to embryonated chicken eggs [5,6]. HAZV is therefore considered to be a good surrogate model for CCHFV that can be used safely.

HAZV is an enveloped virus, and its genome contains three segments of single-stranded, negative-sense RNA (-ssRNA) including the 1677 nucleotide long S (small; encoding nucleoprotein (N)), 4575 nucleotide long M (medium; glycoprotein (Gn and Gc)) and 11,980 nucleotide long L genes (large; RNA-dependent RNA polymerase (RdRp)) [7]. HAZV N protein is structurally similar to CCHFV N protein, and their amino acid sequences are approximately 60% identical [8,9,10]. N protein encapsidates genomic and anti-genomic viral RNA, which are transcribed and replicated by RdRp. HAZV N protein seems to be multifunctional. It binds to heat shock protein 70 for efficient growth of HAZV [11,12]. Furthermore, there has been demonstration of the potential of N protein to interact with several host proteins [13]. N protein binds to the tripartite motif-containing protein 25 (TRIM25) to inhibit type I IFN production [14]. More recently, we also found that N protein interacts with claudin-1 to facilitate virus cell-to-cell spread [15].

Transcriptome analyses of CCHFV have been performed by various groups. The expression patterns of many genes have been shown to be altered by CCHFV infection [16,17,18]. Up-regulation has been shown in many genes, including cytokines and IFN-related genes, seemingly resulting from host response against virus infection. The modulation of host gene expression other than innate immunity could also be advantageous to the virus.

In the current study, we performed HAZV transcriptome analysis to investigate the alterations of gene expression by HAZV infection. We hypothesized that multifunctional HAZV N protein greatly influences gene expression profiles of HAZV-infected cells. Thus, we especially focused upon the regulation of gene expression by HAZV N protein using a cell line constitutively expressing N protein. Our study revealed potential candidates for host factors affected by HAZV N protein.

## 2. Materials and Methods

### 2.1. Cells and Virus

SW13 cells (kindly provided by Prof. Jiro Yasuda) were grown in Dulbecco’s modified Eagle’s minimal essential medium containing 5% fetal calf serum. A SW13 cell line constitutively expressing HAZV N protein (SW13/N) was previously described [19]. Briefly, SW13 cells were transfected with a pcDNA3.1 vector (Thermo Fisher Scientific, Waltham, MA, USA, Cat# V79020) carrying the HAZV N gene and selected in the presence of Hygromycin B (Wako, Osaka, Japan, Cat# 084-07681) until colonies were visible. Each clone was analyzed for expression levels of N protein, and the clone with high protein expression was used as SW13/N. All cells were maintained in a humidified incubator at 37 °C with 5% CO_2_. We used HAZV (strain JC280, kindly provided by Prof. Roger Hewson) in this study [20]. For virus stock preparation, SW13 cells were infected with HAZV, and the supernatants were aliquoted and stored at −80 °C until use.

### 2.2. Antibodies and Reagent

A monoclonal antibody against HAZV N protein (911-1) has been previously described [19]. Anti-myeloid differentiation factor 88 (MyD88) and actin monoclonal antibodies were purchased from Cell Signaling Technology (Danvers, MA, USA, Cat# 4283S) and Wako (Cat# 010-27841), respectively. Human IFN-α was purchased from Mochida Chemical Industries (Osaka, Japan, production discontinued).

### 2.3. Microarray Analysis

SW13 cells and SW13/N with similar passages were infected with HAZV at an MOI of 0.1 for 48 h. Total RNA was extracted from these cells using Isogen (Nippon Gene Co. Ltd., Tokyo, Japan, Cat# 311-02501) according to the manufacturers’ instructions. Microarray analysis was performed by Filgen, Inc. (Nagoya, Japan). Briefly, purified RNA was labeled using the GeneChip™ WT Plus Reagent Kit (Thermo Fisher Scientific, Cat# 902280), then hybridized to a Clariom™ S Assay, Human (Thermo Fisher Scientific, Cat# 902926). The array strips were analyzed using GeneChip™ Scanner 3000 7G (Thermo Fisher Scientific, Cat# 00-0213). Data were validated using Transcriptome Analysis Console™ v 4.0.3 (Thermo Fisher Scientific) and subjected to normalization using the Signal Space Transformation-Robust Multiarray Analysis. Microarray analysis was performed three times using independently prepared total RNA. Comparison of gene expression values between the groups was performed using Microarray Data Analysis Tool v 3.2 (Filgen). Entities with |log2 fold change (FC)| > 1 and *p* < 0.05 were considered significant. *p* values were calculated by comparing gene expression values from three independent experiments using Student’s *t*-test. To exclude data with low reliability, we excluded genes with values below the average expression value of negative control probes. Data were also subjected to gene ontology (GO) analysis and pathway analysis by Microarray Data Analysis Tool v 3.2, which uses the NCBI BioSystems database.

### 2.4. Plaque Assay

SW13 cells and SW13/N were infected with HAZV at an MOI of 0.1 for 24 h and 48 h, and then the supernatants were collected. SW13 cells grown in 12-well plates were infected with the supernatants diluted serially 10-fold in DMEM without FCS. After 1 h of incubation, the virus-containing medium was removed, and then cells were cultured in DMEM containing 2% FCS, 0.25% SeaKem ME agarose and 0.25% SeaPlaque agarose (FMC Bioproducts, Rockland, ME, USA, Cat# SeaKem ME agarose: 50010, SeaPlaque agarose: 50100) until plaques were visible. The cells were then stained with 0.05% neutral red (Wako, Cat# 140-00932) for 24 h. The plaques were then counted. All experiments were performed in duplicate.

### 2.5. Quantitative Real-Time Reverse Transcription PCR (RT-qPCR)

Total RNA was extracted from these cells using Isogen. cDNA was synthesized using a PrimeScript RT reagent kit (Takara, Shiga, Japan, Cat# RR037A) with oligo-dT primer. cDNAs were then subjected to RT-qPCR using Brilliant III Ultra-Fast SYBR Green Master Mix (Agilent Technologies Inc., Santa Clara, CA, USA, Cat# 600882). The primers used for RT-qPCR were: IFN-induced proteins with tetratricopeptide repeat 1 (*IFIT1*) (F: 5′-CCGAAGAAAAGGCTCTGTGG-3′, R: 5′-TAGGCTGCCCTTTTGTAGCC-3′), IFN lambda 2 (*IFNL2*) (F: 5′-CACCGCTGACACTGACCC-3′, R: 5′-GGTACAGCCAATGGTGGAGG-3′), tissue factor pathway inhibitor 2 (*TFPI2*) (F: 5′-TGTCTCCTGCCCCTAGACTAC-3′, R: 5′-CAAGCATCGTCGCAAGCC-3′), *MyD88* (F: 5′-GACATCCAGTTTGTGCAGGAG-3′, R: 5′-GGCCTTCTAGCCAACCTCTTT-3′) and glyceraldehyde-3-phosphate dehydrogenase (*GAPDH*) (F: 5′-GAAGGTCGGAGTCAACGGATTT-3′, R: 5′-ATCTTGAGGCTGTTGTCATACTTCT-3′). GAPDH was used as an internal control.

### 2.6. Immunoblot Assay

SW13 cells were lysed by lysis buffer containing 50 mM Tris-HCl (pH7.4), 150 mM NaCl and 0.6% NP-40 and then centrifuged. The cell lysates were separated by SDS-PAGE and transferred to Amersham™ Protran™ 0.45 μm nitrocellulose membranes (Cytiva, Marlborough, MA, USA, Cat# 10600007). The membranes were reacted with appropriate primary antibodies, followed by the reaction with peroxidase enzyme-conjugated secondary antibodies (Vector laboratories, Newark, CA, USA, Cat# anti-mouse IgG: PI-2000, anti-rabbit IgG: PI-1000)). The immobilized proteins were detected by luminol reagent (Santa Cruz Biotechnology, Paso Robles, CA, USA, Cat# sc-2048).

## 3. Results

### 3.1. HAZV Infection Up-Regulated Many Immune-Response-Related Genes

**Figure 1:**
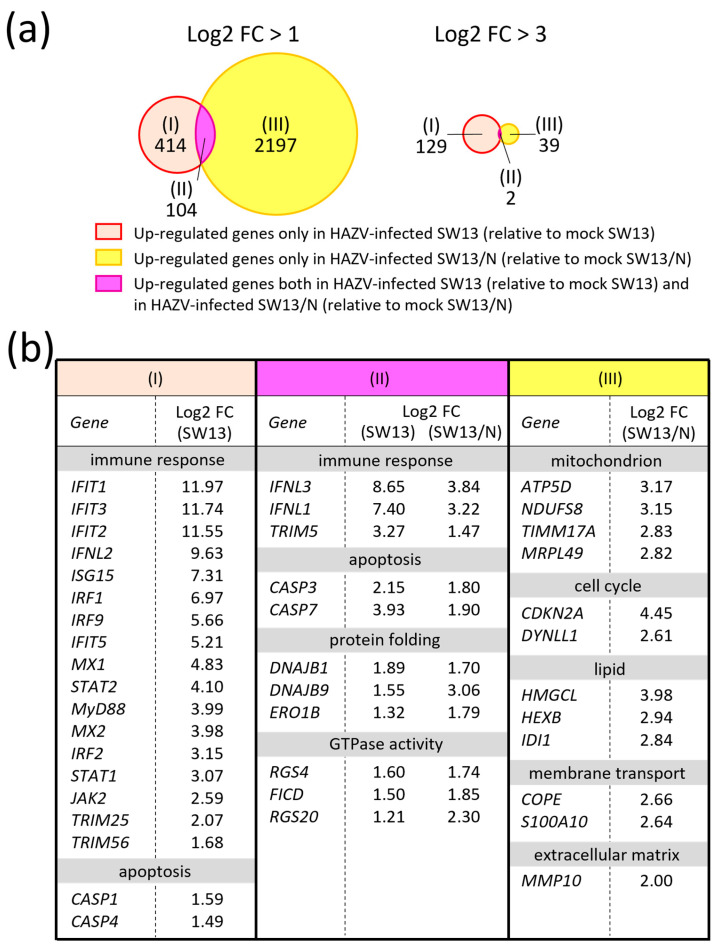
Comparison of up-regulated genes in HAZV-infected SW13 cells and HAZV-infected SW13/N using microarray analysis. (**a**) Venn diagram of up-regulated genes in HAZV-infected SW13 cells (relative to mock SW13 cells) ((I) + (II)) and in HAZV-infected SW13/N (relative to mock SW13/N) ((II) + (III)) with log2 FC > 1 (left) and log2 FC > 3 (right). The Venn diagram was created using the BioInfoRX Venn diagram plotter (https://bioinforx.com/apps/venn.php (accessed on 17 December 2025)). The numbers in the Venn diagram indicate gene counts. (**b**) Gene expression profiles of representative up-regulated genes. Log2 values are shown as the means of three independent experiments. Genes are classified into (I), (II) and (III) as in (**a**).

We compared the gene expression profile between mock SW13 cells and HAZV-infected SW13 cells at an MOI of 0.1 by Clariom S microarray analysis. We wanted to perform infection at a higher MOI, but the highest possible MOI was 0.1 because of the low titer of virus stock. We previously reported that SW13 cells are detached by HAZV infection beyond 48 h post-infection (hpi) [21]. Thus, HAZV-infected cells at 48 hpi were used for the analysis. Out of 24,531 genes, HAZV infection increased expression levels of 518 genes at least two-fold (log2 FC > 1, *p* < 0.05) (Figure 1a, (I) + (II), left). It is noted that some genes with nearly *p* = 0.05 may be false positives in all microarray data, because we could not perform false discovery rate correction. There were 131 genes whose expression levels were increased by HAZV infection in SW13 cells with log2 FC values > 3 (Figure 1a, (I) + (II), right). GO/Pathway analyses revealed that the up-regulated genes were involved in host response to the virus, such as IFN signaling and cytokine signaling (Table 1). Notably, *IFIT genes*, including *IFIT1*, *IFIT2* and *IFIT3*, showed high log2 FC values (log2 FC > 10) (Figure 1b, (I)). GO analysis demonstrated that many genes involved in nucleic acid synthesis and modification were also up-regulated (Table 1).

### 3.2. HAZV N Protein Blocks Expression of Immune-Response-Related Genes

**Figure 2:**
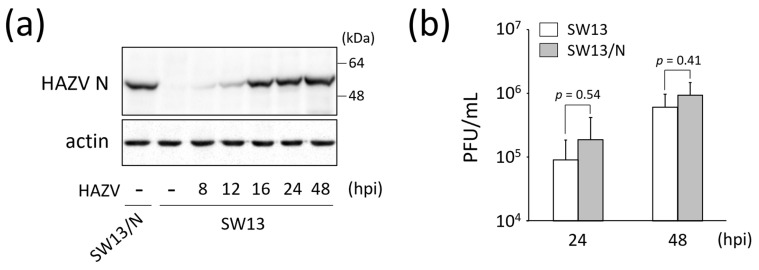
Characterization of SW13/N. (**a**) SW13 cells were infected with HAZV at an MOI of 0.1 for the indicated times. The lysates of these cells and SW13/N were subjected to immunoblot using anti-HAZV N mAb. Actin was used for a loading control. (**b**) SW13 cells and SW13/N were infected with HAZV at an MOI of 0.1 for 24 and 48 h, and the virus titers in the supernatant were determined by plaque assay. PFU/mL values are shown as the means from three independent experiments. *p* values were calculated by the Student’s *t*-test. Error bars indicate standard deviations.

To investigate the effects of HAZV N protein on the transcriptome of HAZV-infected cells, we used an SW13 cell line constitutively expressing HAZV N protein (SW13/N) (Figure 2a) [19]. The amount of N protein in SW13/N was similar to that in HAZV-infected SW13 cells at 16 hpi (Figure 2a). The virus titers in the supernatant of SW13/N were slightly higher than those of SW13 cells at both 24 and 48 hpi, but without significance (Figure 2b). We compared the gene expression profiles of HAZV-infected SW13/N at 48 hpi with those of mock SW13/N. Many (n = 2301) genes were up-regulated more than two-fold in HAZV-infected SW13/N, relative to mock SW13/N (log2 FC > 1, *p* < 0.05) (Figure 1a, (II) + (III), left). However, the number of genes with log2 FC > 3 was restricted to only 41 (Figure 1a, (II) + (III), right). The gene expression profiles of HAZV-infected SW13/N appeared to be quite different from those of HAZV-infected SW13 cells. Notably, many immune-response-related genes, including *MyD88*, IFN regulatory factors (*IRFs*) and signal transducer and activator of transcriptions (*STATs*), were up-regulated by HAZV infection only in SW13 cells and not in SW13/N (Figure 1b, (I)). Only two genes, *IFNL1* and *IFNL3*, were up-regulated both in SW13 cells and in SW13/N with log2 FC values > 3 (Figure 1a, (II), right and Figure 1b). However, the log2 FC values of these genes in SW13/N were much lower than those in SW13 cells (Figure 1b, (II)). This clearly indicates the suppression of expression of these genes by N protein. N protein also appears to suppress HAZV-induced gene expression of apoptosis-inducer caspase, caspase-7 (*CASP7*) (Figure 1b, (II)). 

Log2 FC values of DnaJ (Hsp40) homolog subfamily B member 9 (*DNAJB9*) and regulator of G-protein signaling 20 (*RGS20*) in SW13/N were higher than those in SW13 cells (Figure 1b, (II)). Expression of these genes is possibly promoted by N protein in infected cells. Log2 FC values of *DNAJB1*, endoplasmic reticulum oxidoreductase beta (*ERO1B*), *RGS4* and FIC domain containing (*FICD*) in SW13 cells were similar to those in SW13/N (Figure 1b, (II)). N protein seemingly does not affect the expression level of these genes. Out of the 104 genes shown in Figure 1a ((II), left), 23 genes (such as *IFNL3*) showed higher log2 FC values in SW13 cells than in SW13/N, and 13 genes (such as *DNAJB9*) showed lower log2 FC values in SW13 cells than in SW13/N. The remaining 68 genes (such as *ERO1B*) showed similar log2 FC values in SW13 cells and in SW13/N.

GO/Pathway analyses revealed that HAZV infection in SW13/N did not statistically up-regulate the immune-response-related genes but rather the genes of the mitochondrion and respiratory electron transport (Table 2). The genes related to the mitochondrial electron transport system, such as ATP synthase, H+ transporting, mitochondrial F1 complex delta subunit (*ATP5D*) and NADH:ubiquinone oxidoreductase core subunit S8 (*NDUFS8*), were up-regulated (Figure 1b, (III)). Other up-regulated genes were involved in the cell cycle, lipids and membrane transport (Figure 1b, (III)).

### 3.3. HAZV Infection Down-Regulates Cell-Cycle- and Cytoskeleton-Related Genes

**Figure 3:**
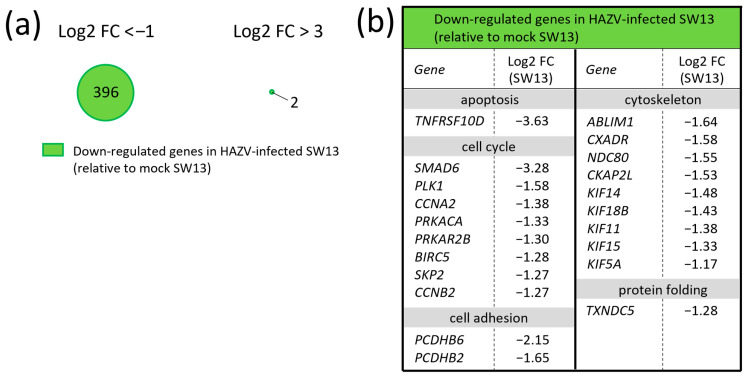
Down-regulated genes by HAZV infection in SW13 cells. (**a**) Down-regulated genes in HAZV-infected SW13 cells (relative to mock SW13 cells) with log2 FC < −1 (left) and log2 FC < −3 (right). The numbers in the circles indicate gene counts. (**b**) Gene expression profiles of representative down-regulated genes. Log2 values are shown as the means of three independent experiments.

The expression levels of 396 genes were down-regulated less than 0.5-fold by HAZV infection (log2 FC < −1, *p* < 0.05) (Figure 3a, right). Many genes related to regulation of cell cycle and cytoskeleton were down-regulated (Figure 3b and Table 3), which seems to be the result of manipulation by viruses, because viruses regulate the host cell cycle and cytoskeleton to facilitate viral growth [9,22,23,24]. Most genes down-regulated by HAZV infection in SW13 cells showed moderate log2 FC values in the range of −2 to −1 (Figure 3b). Only two genes, tumor necrosis factor receptor superfamily member 10d (*TNFRSF10D*) and SMAD family member 6 (*SMAD6*), showed log2 FC values < −3 in HAZV-infected SW13 cells (Figure 3b). We also investigated the effects of N protein on HAZV-induced down-regulation of genes. Unexpectedly, no genes were significantly down-regulated in HAZV-infected SW13/N relative to mock SW13/N (Figure 3a).

### 3.4. Various Genes Are Strongly Down-Regulated Both in Mock SW13/N and in HAZV-Infected SW13/N to a Similar Extent

**Figure 4:**
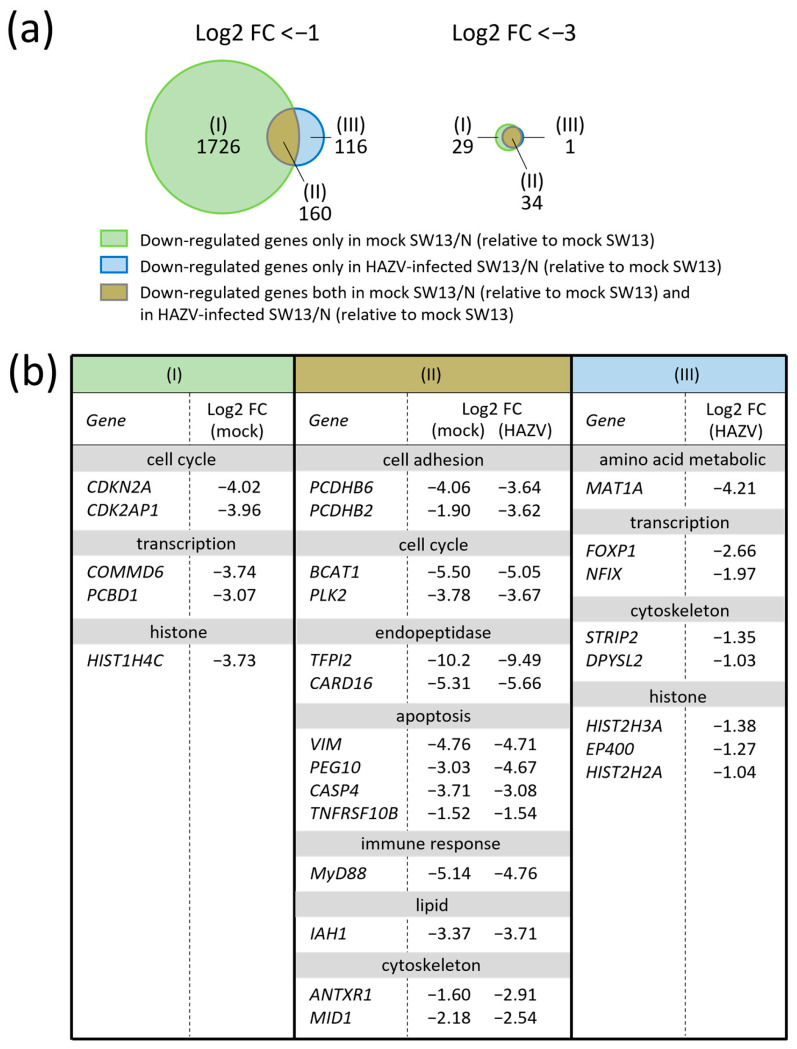
Comparison of down-regulated genes in mock SW13/N cells and HAZV-infected SW13/N using microarray analysis. (**a**) Venn diagram of down-regulated genes in mock SW13/N (relative to mock SW13 cells) ((I) + (II)) and in HAZV-infected SW13/N (relative to mock SW13 cells) ((II) + (III)) with log2 FC < −1 (left) and log2 FC < −3 (right). The Venn diagram was created using the BioInfoRX Venn diagram plotter (https://bioinforx.com/apps/venn.php (accessed on 17 December 2025)). The numbers in the Venn diagram indicate gene counts. (**b**) Gene expression profiles of representative down-regulated genes. Log2 values are shown as the means of three independent experiments. Genes are classified into (I), (II) and (III) as in (**a**).

We hypothesized that many genes are down-regulated even in mock SW13/N and are not further down-regulated in HAZV-infected SW13/N. To assess this possibility, we compared the gene expression profiles of mock SW13/N with those of mock SW13 cells. As a result, 1886 genes were down-regulated in mock SW13/N, relative to mock SW13 cells, with log2 FC < −1 (Figure 4a, (I) + (II), left). Log2 FC < −3 was shown in 63 genes (Figure 4a, (I) + (II), right). Next, we compared the gene expression profiles between mock SW13 cells and HAZV-infected SW13/N. In total, 276 genes were down-regulated < 0.5-fold in HAZV-infected SW13/N, relative to mock SW13 cells (Figure 4a, (II) + (III), left). Among them, 160 genes were down-regulated both in HAZV-infected SW13/N and in mock SW13/N (Figure 4a, (II), left). Notably, most genes down-regulated in HAZV-infected SW13/N with log2 FC values < −3 were also down-regulated in mock SW13/N (Figure 4a, (II), right). Furthermore, most genes shown in Figure 4b, (II) are suggested to be strongly down-regulated both in mock SW13/N and in HAZV-infected SW13/N to a similar extent.

The log2 FC value of the *TFPI2 gene* was the lowest in both mock SW13/N (−10.2) and in HAZV-infected SW13/N (9.49) (Figure 4b, (II)). Branched chain amino acid transaminase 1 (*BCAT1*), polo-like kinase 2 (*PLK2*), isoamyl-acetate hydrolyzing esterase 1 (*IAH1*) and several apoptosis-related genes also showed low log2 FC values in SW13/N (Figure 4b, (II)). Surprisingly, *MyD88* showed the low log2 FC value of −5.14 in mock SW13/N and of −4.76 in HAZV-infected SW13/N (Figure 4b, (II)), even though this gene was up-regulated by HAZV infection in SW13 cells (Figure 1b, (I)).

The genes listed in Figure 4b, (I) were down-regulated only in mock SW13/N, not in HAZV-infected SW13/N. The genes listed in Figure 4b, (III) were down-regulated only in HAZV-infected SW13/N, not in mock SW13/N. Evaluating the effects of N protein on the expression of these genes is difficult.

### 3.5. N Protein Down-Regulates MyD88 Expression at Protein Level

**Figure 5:**
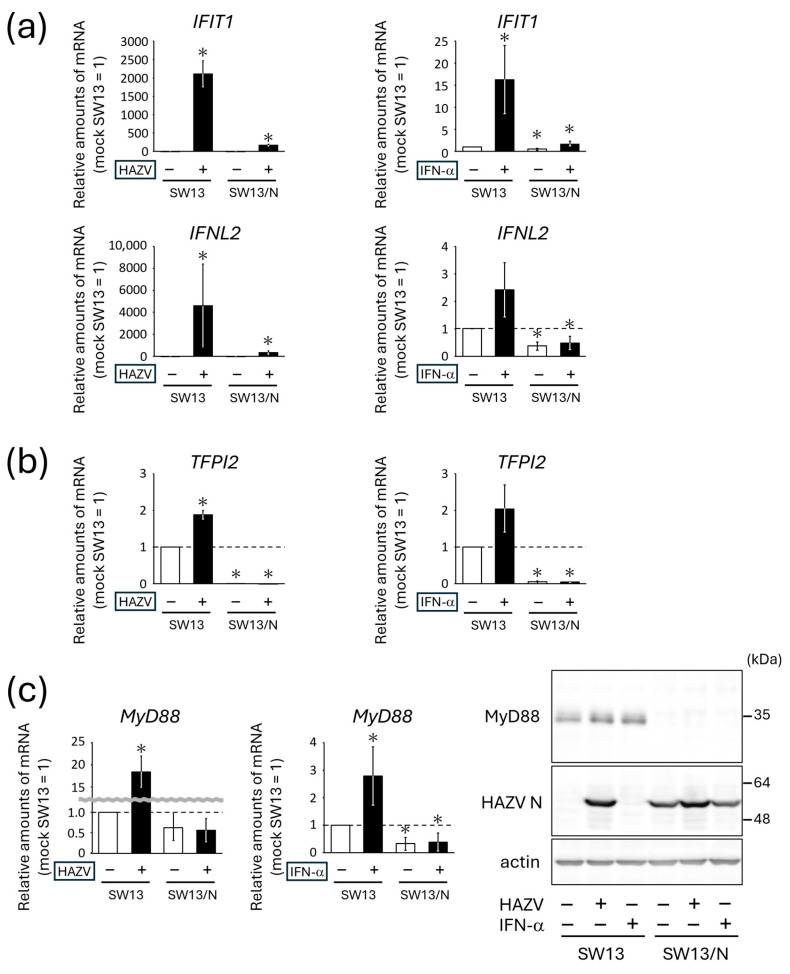
Effects of N protein on the host gene and protein expression. (**a**,**b**) SW13 cells and SW13/N were infected with HAZV at an MOI of 0.1 for 48 h or were treated with 1000 U/mL IFN-α for 48 h. Expression of the indicated mRNA was measured by RT-qPCR, as described in the Materials and Methods section. Data are the means from three independent experiments and are shown as the relative value (mock SW13 cells = 1). *p* values were calculated by the Student’s *t*-test. * *p* < 0.05, compared to values of mock SW13 cells. Error bars indicate standard deviations. (**c**) The cells were subjected to RT-qPCR as described in (**a**). The cells were also subjected to immunoblot analysis using the indicated Abs. Actin was used as a loading control. The immunoblot was performed three times independently, and the representative data are shown.

To verify the results of microarray analysis, the expression levels of differentially expressed genes were quantified by RT-qPCR. As shown in Figure 1b, gene expression of *IFIT1* and *IFNL2* was strongly induced in HAZV-infected SW13 cells, while the induction was moderate in HAZV-infected SW13/N. Similarly, mRNA expression level of *IFIT1* and *IFNL2* in HAZV-infected SW13 cells was much higher than that in HAZV-infected SW13/N (Figure 5a).

The amounts of *TFPI2 mRNA* in HAZV-infected SW13 cells were less than two-fold higher than those in mock SW13 cells (Figure 5b), consistent with the results of microarray analysis that the *TFPI2 gene* was moderately up-regulated in HAZV-infected SW13 cells (log2 FC value of 0.47). The *TFPI2 gene* was prominently down-regulated both in mock SW13/N and in HAZV-infected SW13/N (Figure 4b, (II)). RT-qPCR also showed that *TFPI2 mRNA* expression was almost never detected in mock SW13/N (Figure 5b). HAZV infection did not affect the expression of *TFPI2 mRNA* in SW13/N (Figure 5b), probably because of poor expression levels of *TFPI2 mRNA* in SW13/N.

Although the *MyD88 gene* was up-regulated in HAZV-infected SW13 cells (Figure 1b, (I)), it was down-regulated in mock SW13/N and HAZV-infected SW13/N to a similar extent (Figure 4b, (II)). *MyD88 mRNA* expression was induced in HAZV-infected SW13 cells (Figure 5c). The amounts of *MyD88 mRNA* in mock SW13/N were lower than those in mock SW13 cells (Figure 5c). HAZV infection did not affect the amounts of *MyD88 mRNA* in SW13/N (Figure 5c). These results are consistent with the microarray results (Figure 4b, (II)). In addition, we investigated whether N protein affects MyD88 expression at protein levels using immunoblot. Surprisingly, MyD88 protein was not detected at all in either mock SW13/N or in HAZV-infected SW13/N (Figure 5c).

## 4. Discussion

We investigated the alteration of gene expression by HAZV infection. As expected, HAZV infection caused induction of several IFN-stimulated genes (ISGs), including *IFIT1*, myxovirus resistance 1 (*Mx1*) and IFN stimulated gene 15 (*ISG15*) (Figure 1b, (I)), all of which were also induced by CCHFV infection [18]. CCHFV infection has been reported to uniquely induce three genes of type III IFNs (*IFNL1*, *IFNL2* and *IFNL3*) [18]. These genes were also stimulated by HAZV infection (Figure 1b, (I) and (II)). HAZV and CCHFV infection are suggested to induce similar host immune responses.

HAZV infection up-regulates or down-regulates numerous genes (Figure 1a, (I) + (II) and Figure 2a). Determining the genes in which the expression is affected by N protein by simply infecting SW13 cells is therefore difficult. To focus on the effects of N protein, we used a cell line that constitutively expresses N protein (SW13/N), although we recognize that N protein expressed by transfection is not necessarily the same as N protein in infected cells. It should be noted that some cellular genes/proteins might be affected by the expression of other cellular genes/proteins, not directly related to the influence of N protein. HAZV infection up-regulated immune-response-related genes including *STAT1*, *STAT2* and Janus kinase 2 (*JAK2*) in SW13 cells only, not in SW13/N (Figure 1b, (I)). N protein is therefore clearly indicated to inhibit expression of these genes, which is one of our findings. *IFIT1*, *IFNL2*, *TFPI2* and *MyD88 genes* and MyD88 protein that were up-regulated by HAZV infection were also up-regulated by IFN-α treatment in SW13 cells, but not in SW13/N (Figure 5). N protein might inhibit the expression of these genes by suppressing the IFN signaling pathway. Immune response is likely to be immediately activated in SW13 cells in response to HAZV infection. The activation is inhibited in HAZV-infected SW13/N, indicating the inhibition of induction of an anti-viral state in SW13/N.

We recently demonstrated that N protein binds to TRIM25 to inhibit type I IFN production [14]. N protein therefore seems to inhibit both type I IFN production and the IFN signaling pathway. Interestingly, gene expression of *MyD88* was uniquely reduced in SW13/N (Figure 4b, (II)), despite its up-regulation in HAZV-infected SW13 cells (Figure 1b, (I)), an indication of no induction of the *MyD88 gene* by N protein. Furthermore, MyD88 protein was not detected at all in SW13/N (Figure 5c). Another two SW13/N cell lines also did not show expression of MyD88 protein. Thus, there is a possibility that MyD88 protein might be degraded by N protein. MyD88 protein is reportedly degraded by some viral proteins, such as ICP0 protein of HSV and RTA protein of Kaposi’s-sarcoma-associated herpesvirus [25,26,27]. We are currently continuing to investigate the relationship between HAZV N protein and MyD88 protein.

The genes listed in Figure 1b, (III) were up-regulated in HAZV-infected SW13/N, but not in HAZV-infected SW13 cells. N protein might directly affect the expression levels of these genes. However, it should be noted that the expression profiles of mock SW13/N were quite different from those of mock SW13 cells (Figure 4a, (I) + (II)). Therefore, a more feasible explanation is that the up-regulation of the genes shown in Figure 1b, (III) might have been caused by alteration of the expression pattern of some other gene(s) in SW13/N. The matrix metallopeptidase 10 (*MMP10*) gene was up-regulated only in HAZV-infected SW13/N (Figure 1b, (III)), while *TFPI2* was strongly down-regulated in SW13/N (Figure 4b, (II)). *TFPI2* reportedly inhibits expression of MMPs including *MMP10* [28]. Up-regulation of the *MMP10 gene* in SW13/N might therefore result from down-regulation of the *TFPI2 gene*.

We also examined down-regulated genes by N protein. No genes were significantly down-regulated in HAZV-infected SW13/N, relative to mock SW13/N, so we compared the gene expression profiles of mock or HAZV-infected SW13/N with those of mock SW13 cells. GO analysis showed that many genes involved in protein synthesis (including translation, mRNA processing, rRNA binding, etc.) were down-regulated in mock SW13/N, relative to mock SW13 cells (Table 4). Down-regulated genes in HAZV-infected SW13/N, relative to mock SW13 cells, were chromatin- and histone-related genes with functions of DNA regulation and maintenance (Table 5). Down-regulated genes in HAZV-infected SW13/N and in mock SW13/N therefore seem to be different. However, many genes down-regulated in HAZV-infected SW13/N were also down-regulated in mock SW13/N, in particular with log2 FC values < −3 (Figure 4a, (II), right). The log2 FC values in HAZV-infected SW13/N were as low as those in mock SW13/N (Figure 4b, (II)). Expression of these genes is potentially inhibited by N protein. The expression level of these genes might be extremely low in SW13/N, and never decreased by HAZV infection, but the possibility of involvement of HAZV infection in expression of these genes could not be ruled out. This may be a reason why no genes were significantly down-regulated in HAZV-infected SW13/N, relative to mock SW13/N. Log2 FC values of protocadherin beta 2 (*PCDHB2*), paternally expressed gene 10 (*PEG10*) and anthrax toxin receptor 1 (*ANTXR1*) in HAZV-infected SW13/N (relative to mock SW13 cells) were lower than those in mock SW13/N (relative to mock SW13 cells) (Figure 4b, (II)). These genes also showed log2 FC values < −1 in HAZV-infected SW13/N, relative to mock SW13/N, but without significance (*p* > 0.05). We therefore did not include them among the genes that were significantly down-regulated in HAZV-infected SW13/N, relative to mock SW13/N.

The vimentin (*VIM*) gene was down-regulated in mock and HAZV-infected SW13/N to a similar extent (Figure 4b, (II)). SW13 cells exist in two subtypes: SW13+, which expresses VIM, and SW13-, which does not [29,30]. SW13- also does not express either brahma (BRM) or brahma related gene 1 (BRG1) [30,31]. BRM and BRG1 are essential for the BRG1/BRM-associated factor (BAF) chromatin remodeling complex that promotes IFN-inducible genes [32,33,34]. The activity of promotion of IFN-inducible genes may therefore be dependent on the subtypes of SW13 cells. Transition between SW13+ and SW13- can occur after over 20 doublings [35]. Both SW13 cells and SW13/N used in this study were probably heterogeneous mixtures of these subtypes because these cells were passaged over 30 times. Furthermore, the expression level of *BRM* and *BRG1* genes in mock SW13/N was similar to that in mock SW cells. N protein is therefore thought to be unlikely to regulate IFN-inducible genes via BAF function.

Interestingly, the *CASP4 gene* was up-regulated in HAZV-infected SW13 cells but down-regulated in mock SW13/N and HAZV-infected SW13/N, similar to the case with *MyD88* (Figure 1b (I) and Figure 4b (II)). Further investigations are now in progress. It is also noted that *PCDHB2* and *PCDHB6 genes* were down-regulated not only in mock SW13/N and HAZV-infected SW13/N (Figure 4b, (II)) but also in HAZV-infected SW13 cells (Figure 3b). The down-regulation of these genes in HAZV-infected SW13 cells was less pronounced than that in mock SW13/N and HAZV-infected SW13/N (Figure 3b and Figure 4b, (II)). This might be because N protein is not present at first in HAZV-infected SW13 cells, while it is constitutively expressed in SW13/N. The investigations using N-inducible SW13 cell lines such as the Tet-inducible system may lead to the validation of the results.

Viruses use a cytoskeleton, such as actin and tubulin, for their intracellular transport. Cytoskeleton-related genes, including *ANTXR1* and midline 1 (*MID1*), were down-regulated in SW13/N (Figure 4b, (II)). Another cytoskeleton-related gene, dihydropyrimidinase-like 2 (*DPYSL2*), was down-regulated in HAZV-infected SW13/N (Figure 4b, (III)). N protein possibly manipulates expression of these cytoskeleton-related genes to promote intracellular transport of HAZV. DPYSL2 protein reportedly interacts with HAZV N protein [13]. This interaction might also contribute to regulation of host cell cytoskeletal structures in infected cells, which facilitates intracellular transport of viral components. Detailed binding experiments of N protein with ANTXR1, MID1 and DPYSL2 proteins might reveal potential roles of N protein in the cytoskeleton.

Two cell-adhesion-related genes, *PCDHB2* and *PCDHB6*, were down-regulated in SW13/N (Figure 4b, (II)). Regulation of cell adhesion might be important for virus growth because cell–cell adhesion components are reportedly used as viral receptors or disrupted for bypassing epithelial barriers by viruses [36]. Epithelial cell–cell adhesion is maintained by adherens junctions and tight junctions [37]. To facilitate viral cell-to-cell spread, HAZV N protein reduces cell surface expression of claudin-1, a tight junction protein [15]. N protein might also regulate gene expression of adherens junction molecules to promote cell-to-cell movement of HAZV.

HAZV N protein inhibits apoptosis [19], but the mechanisms remain unknown. Gene expression of apoptosis-inducing caspases, *CASP4* and *CASP7*, was negatively regulated by N protein (Figure 1b, (II) and Figure 4b, (II)). *TFPI2 gene* expression was strongly suppressed in SW13/N (Figure 4b, (II)). TFPI2 reportedly induces apoptosis [38]. N protein might inhibit apoptosis by modulating the gene expression of these apoptosis-related genes. Knockdown experiments using siRNA against these genes might uncover the effects of N protein on apoptosis via regulation of expression of these apoptosis-related genes.

The viral-derived DNA forms (vDNAs) existing in HAZV-infected tick cells reportedly contribute to persistent HAZV infection [39]. This seems specific to tick cells, because vDNAs were not observed in mammalian cells [39]. HAZV polymerase mutations are involved in persistent HAZV infection in human cells [21]. HAZV appears to persistently infect human and tick cells in different ways, indicating the different virus–host cell interactions in these cells. Transcriptome of HAZV in tick cells has not yet been analyzed. Comparison might uncover new insights into the strategies of HAZV for effective growth, including escape from host defense, in both human and tick cells. Furthermore, there are no reports of an in vivo transcriptome of HAZV; in vivo analysis should lead to a better understanding of virus–host interactions in whole organisms.

## 5. Conclusions

HAZV N protein was shown to have great influence on transcriptome of HAZV-infected cells. Structural similarity between N proteins of HAZV and CCHFV suggests their functional similarity. This study might therefore provide new insights into the effects of CCHFV N protein on gene expression profile. This is the first report of comprehensive analysis of host gene expression manipulated by nairovirus protein.

## Figures and Tables

**Table 1:** GO/pathway analyses of genes with increased expression (Log2 FC > 1) (mock SW13 cells vs. infected SW13 cells).

GO Analysis			
Category	Term	Gene Counts ^(^*^)^	*p* Value
biological process	immune system process	69	8.12 × 10^−33^
	cell proliferation	19	8.51 × 10^−3^
cellular component	nucleus	248	4.61 × 10^−14^
	nucleoplasm	132	2.76 × 10^−11^
	intracellular	52	4.16 × 10^−11^
	cytosol	152	4.59 × 10^−10^
	nucleolus	43	4.62 × 10^−4^
	nuclear chromosome	6	8.61 × 10^−3^
molecular function	DNA binding	100	4.36 × 10^−4^
	transcription factor binding	21	1.33 × 10^−3^
	helicase activity	12	1.96 × 10^−3^
	RNA binding	42	2.33 × 10^−3^
	unfolded protein binding	8	1.24 × 10^−2^
	enzyme binding	18	2.32 × 10^−2^
	nuclease activity	8	3.14 × 10^−2^
	ligase activity	20	3.69 × 10^−2^
	nucleotidyltransferase activity	6	4.60 × 10^−2^
Pathway analysis			
Pathways ^(^**^)^		Gene Counts ^(^*^)^	*p* value
IFN signaling		57	9.43 × 10^−35^
cytokine signaling in immune system		93	1.26 × 10^−30^
IFN alpha/beta signaling		36	3.72 × 10^−29^
immune system		144	2.82 × 10^−23^
IFN gamma signaling		29	5.40 × 10^−19^

(*) Sum of gene counts does not match the number of genes shown in Figure 1a because genes may be categorized into overlapping GO terms and pathways. (**) Top 5 pathways with high significance level.

**Table 2:** GO/pathway analyses of genes with increased expression (Log2 FC > 1) (mock SW13/N vs. infected SW13/N).

GO Analysis			
Category	Term	Gene Counts ^(^*^)^	*p* Value
biological process	translation	128	8.08 × 10^−21^
	biosynthetic process	8	2.02 × 10^−20^
	small molecule metabolic process	333	2.31 × 10^−15^
	protein folding	62	2.08 × 10^−9^
	cellular nitrogen compound	57	8.32 × 10^−7^
	metabolic process		2.61 × 10^−2^
	vesicle-mediated transport	63	2.92 × 10^−5^
	ribosome biogenesis	31	6.11 × 10^−5^
	membrane organization	51	1.51 × 10^−3^
	cell cycle	118	1.76 × 10^−3^
	mitosis	59	2.86 × 10^−3^
	mRNA processing	63	7.96 × 10^−3^
	cell division	70	1.04 × 10^−2^
	vacuolar transport	6	1.96 × 10^−2^
	mitochondrion organization	20	2.17 × 10^−2^
cellular component	intracellular	185	1.77 × 10^−75^
	cytoplasm	955	1.07 × 10^−72^
	mitochondrion	473	8.30 × 10^−59^
	cell	16	1.65 × 10^−19^
	cytosol	594	6.00 × 10^−19^
	ribosome	90	1.22 × 10^−16^
	nucleoplasm	470	7.19 × 10^−15^
	nucleus	915	3.08 × 10^−14^
	nucleolus	186	1.96 × 10^−11^
	endoplasmic reticulum	251	3.87 × 10^−8^
	lysosome	70	6.75 × 10^−5^
	vacuole	5	6.90 × 10^−5^
	peroxisome	30	7.68 × 10^−4^
	endosome	91	1.26 × 10^−2^
molecular function	structural constituent of ribosome RNA binding unfolded protein binding structural molecule activity oxidoreductase activity isomerase activity rRNA binding ligase activity GTPase activity	75 160 39 9 109 32 14 72 48	3.50 × 10^−17^ 9.48 × 10^−10^ 4.21 × 10^−9^ 3.48 × 10^−5^ 9.98 × 10^−5^ 4.01 × 10^−4^ 1.37 × 10^−3^ 1.67 × 10^−3^ 3.07 × 10^−3^
Pathway analysis			
Pathways ^(^**^)^		Gene Counts ^(^*^)^	*p* value
metabolism		430	7.90 × 10^−19^
citric acid cycle and		73	5.45 × 10^−16^
respiratory electron transport			
respiratory electron transport, ATP synthesis		61	1.01 × 10^−15^
by chemiosmotic coupling, and heat production			
by uncoupling proteins			
metabolism of proteins		330	1.96 × 10^−15^
mitochondrial translation		53	3.39 × 10^−15^

(*) Sum of gene counts does not match the number of genes shown in Figure 1a because genes may be categorized into overlapping GO terms and pathways. (**) Top 5 pathways with high significance level.

**Table 3:** GO/pathway analyses of genes with decreased expression (Log2 FC < −1) (mock SW13 cells vs. infected SW13 cells).

GO Analysis			
Category	Term	Gene Counts ^(^*^)^	*p* Value
biological process	mitosis	33	2.31 × 10^−13^
	cell cycle	46	3.32 × 10^−13^
	cell division	35	1.04 × 10^−11^
	cell proliferation	23	3.03 × 10^−5^
	chromosome segregation	9	1.79 × 10^−4^
	small molecule metabolic process	50	2.45 × 10^−2^
	cytoskeleton-dependent intracellular transport	2	4.17 × 10^−2^
	DNA metabolic process	3	4.37 × 10^−2^
cellular component	chromosome	35	6.08 × 10^−13^
	cytoskeleton	49	5.50 × 10^−6^
	nuclear chromosome	8	6.03 × 10^−5^
	protein complex	21	5.25 × 10^−4^
	intracellular	26	8.30 × 10^−3^
	cytosol	85	3.51 × 10^−2^
	peroxisome	6	3.82 × 10^−2^
molecular function	ATPase activity	13 9 17 3	4.47 × 10^−4^ 7.55 × 10^−4^ 5.85 × 10^−4^ 1.10 × 10^−2^
	histone binding		
	enzyme binding		
	hydrolase activity, acting on		
	carbon-nitrogen (but not peptide) bonds		
Pathway analysis			
Pathways ^(^**^)^		Gene Counts ^(^*^)^	*p* value
cell cycle, mitotic		44	5.98 × 10^−14^
cell cycle		47	5.17 × 10^−13^
Rho GTPase effectors		29	6.60 × 10^−11^
aurora B signaling		13	7.14 × 10^−11^
M phase		29	1.89 × 10^−10^

(*) Sum of gene counts does not match the number of genes shown in Figure 3a because genes may be categorized into overlapping GO terms and pathways. (**) Top 5 pathways with high significance level.

**Table 4:** GO/pathway analyses of genes with decreased expression (Log2 FC < −1) (mock SW13 cells vs. mock SW13/N).

GO Analysis			
Category	Term	Gene Counts ^(^*^)^	*p* Value
biological process	translation	133	3.30 × 10^−30^
	biosynthetic process	6	4.05 × 10^−29^
	small molecule metabolic process	286	1.54 × 10^−15^
	cellular nitrogen compound	58	6.82 × 10^−10^
	metabolic process		
	ribosome biogenesis	29	9.73 × 10^−6^
	protein folding	43	2.25 × 10^−5^
	mRNA processing	53	8.60 × 10^−3^
	cell division	59	1.09 × 10^−2^
	vacuolar transport	5	2.89 × 10^−2^
	mitosis	45	3.13 × 10^−2^
	cell cycle	90	3.96 × 10^−2^
cellular component	intracellular	165	3.20 × 10^−50^
	mitochondrion	381	1.13 × 10^−47^
	cytoplasm	736	1.88 × 10^−46^
	ribosome	94	5.93 × 10^−24^
	nucleus	768	3.76 × 10^−15^
	nucleolus	170	1.32 × 10^−14^
	nucleoplasm	380	5.35 × 10^−12^
	cell	19	1.25 × 10^−11^
	endoplasmic reticulum	193	3.40 × 10^−5^
	peroxisome	24	2.87 × 10^−3^
	vacuole	4	2.72 × 10^−2^
	lysosome	47	2.96 × 10^−2^
	nuclear envelope	25	3.56 × 10^−2^
molecular function	structural constituent of ribosome	89	1.31 × 10^−30^
	RNA binding	147	1.31 × 10^−13^
	structural molecule activity	7	6.97 × 10^−13^
	unfolded protein binding	27	6.58 × 10^−6^
	oxidoreductase activity	92	5.39 × 10^−5^
	isomerase activity	28	2.49 × 10^−4^
	rRNA binding	12	1.57 × 10^−3^
Pathway analysis			
Pathways ^(^**^)^		Gene Counts ^(^*^)^	*p* value
ribosome		72	1.29 × 10^−23^
metabolism		374	1.40 × 10^−21^
metabolism of amino acids and derivatives		110	1.89 × 10^−19^
translation		69	1.06 × 10^−18^
gene expression		316	2.24 × 10^−18^

(*) Sum of gene counts does not match the number of genes shown in Figure 4a because genes may be categorized into overlapping GO terms and pathways. (**) Top 5 pathways with high significance level.

**Table 5:** GO/pathway analyses of genes with decreased expression (Log2 FC < −1) (mock SW13 cells vs. infected SW13/N).

GO Analysis			
Category	Term	Gene Counts ^(^*^)^	*p* Value
biological process	cell adhesion	19	2.67 × 10^−3^
	cell cycle	19	3.43 × 10^−3^
	cytoskeleton organization	5	4.34 × 10^−2^
cellular component	chromosome	19	3.33 × 10^−6^
	nucleoplasm	56	4.77 × 10^−3^
	proteinaceous extracellular matrix	11	1.79 × 10^−2^
molecular function	DNA binding	56	1.77 × 10^−4^
	helicase activity	6	2.11 × 10^−2^
	histone binding	5	3.11 × 10^−2^
Pathway analysis			
Pathways ^(^**^)^		Gene Counts ^(^*^)^	*p* value
DNA strand elongation		8	2.26 × 10^−7^
telomere maintenance		11	2.39 × 10^−7^
chromosome maintenance		12	4.69 × 10^−7^
cell cycle, mitotic		26	4.88 × 10^−7^
G1/S transition		12	1.59 × 10^−6^

(*) Sum of gene counts does not match the number of genes shown in Figure 4a because genes may be categorized into overlapping GO terms and pathways. (**) Top 5 pathways with high significance level.

## Data Availability

Microarray data in this study are deposited at Gene Expression Omnibus as GSE314331.

## Abbreviations

The following abbreviations are used in this manuscript:

N	nucleoprotein
HAZV	*Orthonairovirus hazaraense*
MyD88	myeloid differentiation factor 88
CCHFV	Crimean-Congo hemorrhagic fever virus
BSL	biosafety level
RdRp	RNA-dependent RNA polymerase
TRIM25	tripartite motif-containing protein 25
FC	fold change
GO	gene ontology
RT-qPCR	quantitative real-time reverse transcription PCR
IFIT	IFN-induced proteins with tetratricopeptide repeat
IFNL	IFN lambda
TFPI2	tissue factor pathway inhibitor 2
GAPDH	glyceraldehyde-3-phosphate dehydrogenase
hpi	hours post-infection
IRF	IFN regulatory factor
STAT	signal transducer and activator of transcription
CASP	caspase
DNAJB9	DnaJ (Hsp40) homolog subfamily B member 9
RGS	regulator of G-protein signaling
ERO1B	endoplasmic reticulum oxidoreductase beta
FICD	FIC domain containing
ATP5D	ATP synthase, H+ transporting, mitochondrial F1 complex delta subunit
NDUFS8	NADH:ubiquinone oxidoreductase core subunit S8
TNFRSF10D	tumor necrosis factor receptor superfamily member 10d
SMAD6	SMAD family member 6
BCAT1	Branched chain amino acid transaminase 1
PLK2	polo-like kinase 2
IAH1	isoamyl-acetate hydrolyzing esterase 1
ISG	IFN-stimulated gene
Mx1	myxovirus resistance 1
JAK	Janus kinase
MMP	matrix metallopeptidase
PCDHB2	protocadherin beta 2
PEG10	paternally expressed gene 10
ANTXR1	anthrax toxin receptor 1
VIM	vimentin
BRM	brahma
BRG1	brahma related gene 1
BAF	BRG1/BRM-associated factor
MID1	midline 1
DPYSL2	dihydropyrimidinase-like 2

## References

[1] Weidmann M., Avsic-Zupanc T., Bino S., Bouloy M., Burt F., Chinikar S., Christova I., Dedushaj I., El-Sanousi A., Elaldi N. (2016). Biosafety standards for working with Crimean-Congo hemorrhagic fever virus. J. Gen. Virol..

[2] Bereczky S., Lindegren G., Karlberg H., Akerström S., Klingström J., Mirazimi A. (2010). Crimean-Congo hemorrhagic fever virus infection is lethal for adult type I interferon receptor-knockout mice. J. Gen. Virol..

[3] Dowall S.D., Findlay-Wilson S., Rayner E., Pearson G., Pickersgill J., Rule A., Merredew N., Smith H., Chamberlain J., Hewson R. (2012). Hazara virus infection is lethal for adult type I interferon receptor-knockout mice and may act as a surrogate for infection with the human-pathogenic Crimean-Congo hemorrhagic fever virus. J. Gen. Virol..

[4] Appelberg S., John L., Pardi N., Végvári Á., Bereczky S., Ahlén G., Monteil V., Abdurahman S., Mikaeloff F., Beattie M. (2022). Nucleoside-modified mRNA vaccines protect IFNAR-/- mice against Crimean-Congo hemorrhagic fever virus infection. J. Virol..

[5] Xia H., Zhao J., Li Y., Yin S., Tang S., Zhang Z., Yu J., Kou Z., Fan Z., Li T. (2013). Infection and propagation of Crimean-Congo hemorrhagic fever virus in embryonated chicken eggs. Virus Res..

[6] Matsumoto Y., Ohta K., Nishio M. (2018). Lethal infection of embryonated chicken eggs by Hazara virus, a model for Crimean-Congo hemorrhagic fever virus. Arch. Virol..

[7] Barr J.N., Weber F., Schmaljohn C.S., Howley P.M., Knipe D.M., Whelan S.P.J. (2020). Bunyavirales. Fields Virology.

[8] Surtees R., Ariza A., Punch E.K., Trinh C.H., Dowall S.D., Hewson R., Hiscox J.A., Barr J.N., Edwards T.A. (2015). The crystal structure of the Hazara virus nucleocapsid protein. BMC Struct. Biol..

[9] Wang W., Liu X., Wang X., Dong H., Ma C., Wang J., Liu B., Mao Y., Wang Y., Li T. (2015). Structural and functional diversity of nairovirus-encoded nucleoproteins. J. Virol..

[10] Ohta K., Saka N., Nishio M. (2024). Identification of critical residues for RNA binding of nairovirus nucleoprotein. J. Virol..

[11] Surtees R., Dowall S.D., Shaw A., Armstrong S., Hewson R., Carroll M.W., Mankouri J., Edwards T.A., Hiscox J.A., Barr J.N. (2016). Heat shock protein 70 family members interact with Crimean-Congo hemorrhagic fever virus and Hazara virus nucleocapsid proteins and perform a functional role in the nairovirus replication cycle. J. Virol..

[12] Tampere M., Pettke A., Salata C., Wallner O., Koolmeister T., Cazares-Körner A., Visnes T., Hesselman M.C., Kunold E., Wiita E. (2020). Novel broad-spectrum antiviral inhibitors targeting host factors essential for replication of pathogenic RNA viruses. Viruses.

[13] Molinas A., Turkina M.V., Magnusson K.E., Mirazimi A., Vikström E. (2017). Perturbation of wound healing, cytoskeletal organization and cellular protein networks during Hazara virus infection. Front. Cell Dev. Biol..

[14] Ohta K., Saka N., Nishio M. (2022). Hazara orthonairovirus nucleoprotein antagonizes type I interferon production by inhibition of RIG-I ubiquitination. Viruses.

[15] Ohta K., Saka N., Fukasawa M., Nishio M. (2023). Hazara orthonairovirus nucleoprotein facilitates viral cell-to-cell spread by modulating tight junction protein, claudin-1. Front. Microbiol..

[16] Arnold C.E., Shoemaker C.J., Smith D.R., Douglas C.E., Blancett C.D., Graham A.S., Minogue T.D. (2021). Host response transcriptomic analysis of Crimean-Congo hemorrhagic fever pathogenesis in the cynomolgus macaque model. Sci. Rep..

[17] Neogi U., Elaldi N., Appelberg S., Ambikan A., Kennedy E., Dowall S., Bagci B.K., Gupta S., Rodriguez J.E., Svensson-Akusjärvi S. (2022). Multi-omics insights into host-viral response and pathogenesis in Crimean-Congo hemorrhagic fever viruses for novel therapeutic target. eLife.

[18] Mo Q., Feng K., Dai S., Wu Q., Zhang Z., Ali A., Deng F., Wang H., Ning Y.J. (2023). Transcriptome profiling highlights regulated biological processes and type III interferon antiviral responses upon Crimean-Congo hemorrhagic fever virus infection. Virol. Sin..

[19] Matsumoto Y., Nouchi T., Ohta K., Nishio M. (2019). Regulation of Hazara virus growth through apoptosis inhibition by viral nucleoprotein. Arch. Virol..

[20] Begum F., Wisseman C.L., Casals J. (1970). Tick-borne viruses of West Pakistan. II. Hazara virus, a new agent isolated from Ixodes redikorzevi ticks from the Kaghan Valley, W. Pakistan. Am. J. Epidemiol..

[21] Ohta K., Saka N., Nishi Y., Nishio M. (2024). Nairovirus polymerase mutations associated with the establishment of persistent infection in human cells. J. Virol..

[22] Bagga S., Bouchard M.J. (2014). Cell cycle regulation during viral infection. Methods Mol. Biol..

[23] Walsh D., Naghavi M.H. (2019). Exploitation of cytoskeletal networks during early viral infection. Trends Microbiol..

[24] Gao X., Chen X., Yu L., Zhao S., Jiu Y. (2025). Host cytoskeleton and membrane network remodeling in the regulation of viral replication. Biophys. Rep..

[25] van Lint A.L., Murawski M.R., Goodbody R.E., Severa M., Fitzgerald K.A., Finberg R.W., Knipe D.M., Kurt-Jones E.A. (2010). Herpes simplex virus immediate-early ICP0 protein inhibits Toll-like receptor 2-dependent inflammatory responses and NF-kappaB signaling. J. Virol..

[26] Zhao Q., Liang D., Sun R., Jia B., Xia T., Xiao H., Lan K. (2015). Kaposi’s sarcoma-associated herpesvirus-encoded replication and transcription activator impairs innate immunity via ubiquitin-mediated degradation of myeloid differentiation factor 88. J. Virol..

[27] Shahnazaryan D., Khalil R., Wynne C., Jefferies C.A., Ní Gabhann-Dromgoole J., Murphy C.C. (2020). Herpes simplex virus 1 targets IRF7 via ICP0 to limit type I IFN induction. Sci. Rep..

[28] Yan W., Han Q., Gong L., Zhan X., Li W., Guo Z., Zhao J., Li T., Bai Z., Wu J. (2022). MBD3 promotes hepatocellular carcinoma progression and metastasis through negative regulation of tumour suppressor TFPI2. Br. J. Cancer.

[29] Sarria A.J., Lieber J.G., Nordeen S.K., Evans R.M. (1994). The presence or absence of a vimentin-type intermediate filament network affects the shape of the nucleus in human SW-13 cells. J. Cell Sci..

[30] Davis M.R., Daggett J.J., Pascual A.S., Lam J.M., Leyva K.J., Cooper K.E., Hull E.E. (2016). Epigenetically maintained SW13+ and SW13- subtypes have different oncogenic potential and convert with HDAC1 inhibition. BMC Cancer.

[31] Dunaief J.L., Strober B.E., Guha S., Khavari P.A., Alin K., Luban J., Begemann M., Crabtree G.R., Goff S.P. (1994). The retinoblastoma protein and BRG1 form a complex and cooperate to induce cell cycle arrest. Cell.

[32] Wang W., Côté J., Xue Y., Zhou S., Khavari P.A., Biggar S.R., Muchardt C., Kalpana G.V., Goff S.P., Yaniv M. (1996). Purification and biochemical heterogeneity of the mammalian SWI-SNF complex. EMBO J..

[33] Cui K., Tailor P., Liu H., Chen X., Ozato K., Zhao K. (2004). The chromatin-remodeling BAF complex mediates cellular antiviral activities by promoter priming. Mol. Cell. Biol..

[34] Letimier F.A., Passini N., Gasparian S., Bianchi E., Rogge L. (2007). Chromatin remodeling by the SWI/SNF-like BAF complex and STAT4 activation synergistically induce IL-12Rbeta2 expression during human Th1 cell differentiation. EMBO J..

[35] Yamamichi-Nishina M., Ito T., Mizutani T., Yamamichi N., Watanabe H., Iba H. (2003). SW13 cells can transition between two distinct subtypes by switching expression of BRG1 and Brm genes at the post-transcriptional level. J. Biol. Chem..

[36] Mateo M., Generous A., Sinn P.L., Cattaneo R. (2015). Connections matter—How viruses use cell-cell adhesion components. J. Cell Sci..

[37] Ivanov A.I., Lechuga S., Marino-Melendez A., Naydenov N.G. (2022). Unique and redundant functions of cytoplasmic actins and nonmuscle myosin II isoforms at epithelial junctions. Ann. N. Y. Acad. Sci..

[38] Jia Y., Yang Y., Brock M.V., Cao B., Zhan Q., Li Y., Yu Y., Herman J.G., Guo M. (2012). Methylation of TFPI-2 is an early event of esophageal carcinogenesis. Epigenomics.

[39] Salvati M.V., Salaris C., Monteil V., Del Vecchio C., Palù G., Parolin C., Calistri A., Bell-Sakyi L., Mirazimi A., Salata C. (2021). Virus-derived DNA forms mediate the persistent infection of tick cells by Hazara virus and Crimean-Congo hemorrhagic fever virus. J. Virol..

